# Supplementary material for: Delineating MYC-Mediated Escape Mechanisms from Conventional and T Cell-Redirecting Therapeutic Antibodies
Source: Int J Mol Sci. 2024 Nov 11;25(22):12094. doi: 10.3390/ijms252212094 (PMC11594070; doi:10.3390/ijms252212094)
Supplement: Supplementary file 1 [file ijms-25-12094-s001.zip › ijms-3243150-supplementary.pdf]

Delineating MYC-mediated escape mechanisms from conventional and T-cell redirecting therapeutic antibodies

A. Vera de Jonge, MD[1,2,*], Tamás Csikós, PhD[1,2], Merve Eken[1,2], Elianne P. Bulthuis, PhD[3], Pino J. Poddighe, PhD[3], Margaretha G.M. Roemer, PhD[1,2], Martine E.D. Chamuleau, MD, PhD[1,2], Tuna Mutis, MD,PhD[1,2]

1 Department of Hematology, Amsterdam UMC Location Vrije Universiteit; Amsterdam, 1081 HV, The Netherlands
2 Cancer Center Amsterdam, Cancer Biology and Immunology; Amsterdam, 1081 HV, The Netherlands
3 Department of Clinical Human Genetics, Amsterdam UMC Location Vrije Universiteit; Amsterdam, 1081 HV, The Netherlands
* corresponding author

Supplementary information:

- Supplementary Methods
- Supplementary Tables S1-S3
- Supplementary Figures S1-S4

Supplementary Methods

Primary patient samples and peripheral blood mononuclear cells from healthy donors

To obtain primary lymphoma cells, lymph node excisions from eight patients were mechanically disrupted into small fragments and cultured in RPMI-1640 (Invitrogen, Carlsbad, CA, USA) supplemented with 10% fetal bovine serum (Invitrogen) and 1% penicillin-streptomycin (Invitrogen, 15140122) at 37˚C, 5% CO_2 for 16 hours. The supernatant was filtered using a 70 µM Easy Strainer (Greiner Bio-one, Alphen a/d Rijn, the Netherlands) to collect the malignant B-cells, T-cells and other cells diffused cells of the biopsy fragments.

Bone Marrow (BM) Mononuclear Cells (BMMC) cells were isolated from BM aspirates obtained from three MM patients. Peripheral Blood Mononuclear Cells (PBMCs) from healthy donors were isolated from buffy coats obtained from Sanquin (Amsterdam, The Netherlands). BMMCs and PBMCs were isolated by Ficoll-Hypaque density-gradient centrifugation. Isolated cells were either used directly or cryopreserved in liquid nitrogen until further use.

Cell lines culture conditions

DLBCL cell lines (OCI-LY18 (ACC 699, RRID:CVCL_1880), WSU-DLCL2 (ACC 779, RRID: CVCL_1902), OCI-LY7 (ACC 688, RRID:CVCL_1881) and SU-DHL-6 (ACC 572, RRID:CVCL_2206)) were purchased from the German Collection of Microorganisms and Cell Cultures (DSMZ). BL cell lines (Daudi (RRID:CVCL_0008) and Raji (RRID:CVCL_0511)) and MM cell lines (MM1.S (RRID:CVCL_8792), RPMI8226 (RRID:CVCL_0014), and U266 (RRID:CVCL_0566)) were previously purchased from the American Tissue Culture Collection (ATCC). DLBCL, BL and MM cell lines were transduced with luciferase (LUC)-GFP as described elsewhere.[59]

The cells were cultured in Iscove's Modified Dulbecco's Medium (IMDM, Gibco, ThermoFisher, 21980032) supplemented with 10% heat-inactivated Fetal Calf Serum (FCS, Sigma-Aldrich, F7524) and 1% penicillin/streptomycin (Invitrogen, 15140122) at 37˚C with 5% CO_2.

LUC-GFP positive MM cell lines were cultured in RPMI-1640 (Gibco, Thermofisher, 524000025)), supplemented with 10% heat-inactivated HyClone Fetal Clone I serum (GE Healthcare Life Sciences, SH30080.03) and 1% penicillin/streptomycin (Invitrogen, 15140122).

HEK293T cells were cultured in Dulbecco's Modified Eagle Medium (DMEM, Gibco, ThermoFisher, 31966-021) supplemented with 10% heat-inactivated FCS and 1%

penicillin/streptomycin. Authenticity of all cell lines was verified by short-tandem repeat (STR) profiling (GenePrint 10 System Promega). Cell lines were used no longer than 4 months and tested negative for mycoplasma contamination during culture and prior to use.

DNA processing reagents, amplification and sequencing

The Q5® High-Fidelity DNA Polymerase and restriction enzymes (REs) were purchased from New England Biolabs and from Thermo Fisher Scientific (Res only). Genomic DNA was isolated from cells using the QIAamp DNA Mini Kit (Qiagen, 51304), whereas DNA fragments were isolated with the NucleoSpin Gel and PCR Clean-up Mini kits (Macherey-Nagel 740609.50). Bacterial cultures (One Shot™ Stbl3™ chemically competent E. coli (Invitrogen, C737303)) transformed with lentiviral plasmids were expanded at 33°C on agar or in liquid cultures at 180 rpm. Plasmids were isolated with the PureLink Hipure Plasmid Filter Maxiprep Kit (Invitrogen, K210017)). DNA sequencing reactions were done using the BigDye™ Terminator v3.1 cycle Sequencing Kit (Applied Biosystems 4336917) and resolved on the 3500 Genetic Analyzer, Applied Biosystems/Hitachi.

CRISPR-Cas9 sgRNA design and cloning

All non-modified primers for generating single guide RNAs (sgRNAs) expression cassettes, PCR and sequencing were purchased from Biolegio BV, Nijmegen The Netherlands.

The primers used to generate the sgRNA expression cassette 1 to target the *MYC*1 site are 5' ACCGGTTCGGGGAGACAACGACGG 3' and its complementary 5' AAACCCGTCGTTGTCTCCCCGAAC 3' and the primers used to generate the sgRNA expression cassette 2 to target the *MYC*2 site, are 5'CACCGTATGACCTCGACTACGACT 3' and its complementary 5'AAACAGTCGTAGTCGAGGTCATAC 3'. The primers used to generate the sgRNA expression cassette 3 for targeting just upstream the *MYC*1 target site are 5' CACCGCTGCTCGCCCTCCTACGTTG 3' and its complementary 5' AAACCAACGTAGGAGGGCGAGCAGC 3'. In these primers, bases in positions 1 to 4 facilitate cloning in the lentiviral sgRNA expression vectors. Each sgRNA expression cassette design contains a G nucleotide in the sense strand immediately followed by the target sequence which is either 19 or 20 bases long.

sgRNA plasmids were isolated and purified using Pure Link^{TM} HiPure Plasmid Maxiprep Kit (Invitrogen, K210017) and cloned in a pLCKO lentiviral backbone (Plasmid #73311, Addgene).
The sgRNA expression cassettes 1 and 2 were cloned into the pLCKO lentiviral sgRNA cassette expression vector.[60]
The pLCKO backbone was a gift from Jason Moffat, Addgene plasmid #73311 ; http://n2t.net/addgene:73311 ; RRID:Addgene_73311.
The sgRNA expression cassette 3 was cloned into the lenti-sgRNA neo backbone,[61] which was a gift from Brett Stringer, Addgene plasmid #104992 ; http://n2t.net/addgene:104992; RRID:Addgene_104992).
The sgRNA expression cassette cloning protocols for these specific vectors are available on the Addgene website.
The packaging plasmids psPAX2 and pMD2.G were gifts from Didier Trono, respectively, Addgene plasmid #12260 ; http://n2t.net/addgene:12260 ; RRID:Addgene_12260 and Addgene plasmid # 12259 ; http://n2t.net/addgene:12259 ; RRID:Addgene_12259.
Lenti-Cas9-2A-Blast DNA vector was a gift from Jason Moffat (Addgene plasmid #73310 ; http://n2t.net/addgene:73310 ; RRID:Addgene_73310).[60]

Lentivirus production

HEK293T cells (RRID:CVCL_0063) were transfected with a mixture of 660 µl reduced serum medium (OPTI-MEM®, Gibco, 31985-062), 4 µg psPAX2 (RRID:Addgene_12260), 4 µg pMD2.G (RRID:Addgene_12259), 48 µl of polyethylenimine (1 mg/ml) (PEI 25K™, linear, MW 25000, transfection grade, Polysciences 9002-98-6, 26913-06-4) and 4 µg of the lentiviral transfer plasmid. Medium was refreshed after 24 hours, and the virus supernatant was harvested 48-72 hours thereafter.

PCR and sequence validation of CRISPR-Cas9 MYC targeting

Genomic DNA was isolated and purified using QIAamp DNA Blood Mini Kit (Qiagen, 51304).
Genotyping was performed by PCR using the primers listed below. PCR products were loaded

on a 1.8% agarose gel. Sanger sequence was performed to confirm established mutations using the following primers.

sgRNA MYC1 forward: CACCGTTCGGGGAGACAACGACGG

sgRNA MYC1 reverse: AAACCCGTCGTTGTCTCCCCGAAC

sgRNA MYC2 forward: CACCGTATGACCTCGACTACGACT

sgRNA MYC2 reverse: AAACAGTCGTAGTCGAGGTCATAC

Oligo/SNP-based genomic array profiling and data analysis

Genomic array profiling was carried out using the CytoSan HD array platform (Affymetrix, Santa Clara, CA). Hybridizations were performed according to the manufacturer's protocols.The data were analyzed using the Chromosome Analysis Suite softwarepackage (Affymetrix), using annotations of genome version GRCh37 (hg19). For a comprehensive interpretation of the oligo/SNP-based genomic array profiling data, we used criteria described by Schoumans et al.[62]

MTT Cell viability assay

Where indicated, the relevant target cells were seeded in a 96-wells flat bottom plate at a density of 8000 cells per well. 10 µL MTT reagent (3-[4,5-dimethylthiazol-2-yl]-2,5-diphenyltetrazolium bromide; Sigma Aldrich) was added and cells were incubated for 4 hours. MTT crystals were dissolved using 150 µL isopropanol-HCl and absorbance was measured at 540 nm using a GloMax Discover Microplate reader (Promega). Values are represented as absolute absorbance (OD).

Flow cytometry-based cytotoxicity assays

Violet tracer (CellTrace^{TM} Violet Cell Proliferation Kit ; ThermoFisher, C34557) labeled target cells were resuspended in IMDM medium (10% FCS and 1% penicillin/streptomycin) and seeded in U-bottomed 96-well plates in 90 µl (15.000 cells per well for cell lines and 20.000 cells/well for primary tumor cells). After incubation of target cells with indicated antibodies as described in the main text, the surviving target cells were enumerated by standard quantitative flow cytometry. Viable cells were determined using LIVE/DEAD Fixable Dead Cell Stain Near-IR fluorescent reactive (Invitrogen) and tumor cells were identified through additional labeling with a defined

set of antibodies listed in the supplement. Flow volume control was done by adding Flow-Count Fluorospheres (Beckman Coulter, 7547053).

The percentage lysis of target cells was calculated according the following formula:

% lysis = 100- % viability

% viability = ((absolute number of surviving cells in treated wells x absolute number of flow count beads) / (absolute number of surviving cells in untreated wells x absolute number of flow count beads) x 100% .

Bioluminescent imaging-based cytotoxicity assays

LUC-GFP-transduced lymphoma or MM cell lines were used as target cells. Where indicated, they were pre-incubated with MYC inhibitor 10058-F4 (12.5-150 µM) for 48 or 96 hours, which is washed away before plating the target cells in white opaque, 96-well flat bottom plates prior to addition of serial dilutions of therapeutic antibodies and adding human PBMCS at indicated effector (PBMC) to target (tumor cell) (E:T) ratios. The luciferase signal produced by surviving tumor cells was determined with a GloMax 96 Microplate Luminometer (Promega) after incubation with 125 µg/mL luciferine (Promega) for 30 minutes at 37°C with 5% CO_2. The percent lysis of target cells calculated according to the following formula: *% lysis = 1 – (absolute number of surviving cells in treated wells) / (absolute number of surviving cells in untreated wells) x 100%*.

Cytokine secretion assays

Indicated cytokines and Granzyme B were measured in cell free supernatants using BD™ Cytometric Bead Array (CB) Human Th1/Th2/Th17 Cytokine Kit (BD Biosciences) and a Granzyme B assay kit (Mabtec 3485-1 H-20) respectively, according manufacturers' protocols.

Flow cytometry-based immunophenotyping

For flow cytometry-based immunophenotyping, suspended sells were stained with fluorochrome conjugated antibodies listed in Supplemental Table 2 in FACS medium (PBS/0.05% Azide/0.1% HSA) and Brilliant Stain Buffer Plus (566385, Becton Dickinson)) for 15 minutes at room temperature. Flow cytometric read out of all assays were performed on a 7-laser BD

LSRFortessa™ (BD Biosciences). To ensure any disturbances were avoided, BD™ CS&T beads were run daily. Compensation beads were used to determine spectral overlap, and compensation was automatically calculated using Diva software. Data were analyzed using FCS Express Flow Cytometry Software (De Novo Software versions 06.0025 and 7.14.0020).

Immunoblotting

Cells were lysed in RIPA lysis buffer (100 mM Tris-HCl, 300 nM NaCl, 10% NP40, 10% sodium deoxycholate, 1% SDS, H_2O) supplemented with Complete Protease Inhibitor cocktail (Roche) for 45 minutes at 4˚C. Protein concentrations of whole cell lysates were determined using Pierce™ BCA Protein Assay Kit (ThermoFisher, 23227). Laemmli's sample buffer (BioRad) with β-mercaptoethanol was added and equalized concentrations of lysates were heated at 95˚C for 5 minutes.

Proteins were separated by 4-20% Mini-PROTEAN® TGX™ Precast Gels (BioRad) and transferred to a pure nitrocellulose membrane (0,45 µm, BioRad, 162-0115). Membranes were blocked with Odyssey blocking buffer (Li-cor biosciences) and incubated with the primary antibody (listed in Supplemental Table 3) over night at 4˚C. After washing with PBS 0.1% Tween, membranes were incubated with secondary antibody dissolved in Odyssey blocking buffer. After washing, proteins were visualized using the Odyssey® Infrared imaging system (Li-cor biosciences). If necessary, membranes were stripped using Restore™ Fluorescent Western Blot stripping buffer (Thermo Scientific, 62300) according manufacturer's instructions.

Supplementary Table S1. Single-nucleotide polymorphisms in OCI-LY18 and MYC targeted clones
Plus (+): aberration present. Minus (-): aberration not present . Percentage: present in mosaic (mos). Number: number of copies.

Aberration	ISCN2020	OCI-LY18 WT	OCI-LY18 Cas9	Clone #1	Clone #2	Clone #3	Clone #4	Clone #5	Clone #6	Clone #7	Clone #8	Clone #9	Clone #10
-Y	arr(Y)x0	+	+	+	+	+	+	+	+	+	+	+	+
del(2)(q33.3q36.1)	arr[GRCh37] 2q33.3q36.1(205701996_221745571)x1	-	-	-	-	+	-	-	-	-	-	-	-
del(2)(q37.1q37.2)	arr[GRCh37] 2q37.1q37.2(232542598_237147544)x1	-	-	-	-	+	-	-	-	-	-	-	-
dup(3)(q22.2q25.1)	arr[GRCh37] 3q22.2q25.1(134067644_152071236)x3	-	-	-	-	-	-	+	-	-	-	-	-
del(3)(q25.1q25.31)	arr[GRCh37] 3q25.1q25.31(151944150_156170257)x1	+	+	+	+	+	+	-	+	+	+	+	+
dup(3)(q25.31q29)	arr[GRCh37] 3q25.31q29(156170365_197851986)x3	-	-	-	-	-	-	+	-	-	-	-	-
del(4)(p15.2p15.1)	arr[GRCh37] 4p15.2p15.1(27097097_30001732)x1	-	-	-	-	-	-	-	-	-	-	+	-
upd(6)	arr(6)x2 hmz	+	+	+	+	+	+	+	+	+	+	+	+
del(7)(q36.1q36.2)	arr[GRCh37] 7q36.1q36.2(151512176_153419287)x1	+	+	+	+	+	+	+	+	+	+	+	+
dup(8)(q24.21q24.3)	arr[GRCh37] 8q24.21q24.3(128754611_146295771)x3	+	+	+	3~4	+	+	+	+	+	+	+	+
upd(9)	arr(9) hmz	mos	-	-	-	-	-	-	-	-	-	-	-
upd dup(9)(p13.1p24.3)	arr[GRCh37] 9p24.3p13.1(203861_38787480)x3 hmz	+	+	+	+	+	+	+	+	+	+	+	+
dup(11)(p15.1p15.5)	arr[GRCh37] 11p15.5p15.1(230615_18857821)x3	46%	-	-	-	-	-	-	-	-	-	-	-
dup(12)(q12q21.1)	arr[GRCh37] 12q12q21.1(38719938_74308181)x3	+	+	+	+	+	+	+	+	+	+	+	+
dup(13)(q12.3q21.32)	arr[GRCh37] 13q12.3q21.32(32180580_68457437)x3	-	-	-	-	-	-	+	-	-	-	-	-
dup(13)(q14.11q14.13)	arr[GRCh37] 13q14.11q14.13(44967593_46236053)x3	+	+	+	+	+	+	-	+	+	+	+	+
dup(13)(q21.32q31.2)	arr[GRCh37] 13q21.32q31.2(67693181_88903338)x3	50%	+	+	+	+	+	4~5	+	+	+	+	+
dup(13)(q31.2q31.3)	arr[GRCh37] 13q31.2q31.3(88906627_92884370)x3	+	~4-5	~4	~4	4~5	4~5	6~7	4~5	4~5	4~5	4~5	~4
del(13)(q31.3q34)	arr[GRCh37] 13q33.1q34(104092713_110681243)x1	50%	+	+	+	+	+	+	+	+	+	+	+
amp(14)(q32.33q32.33)	arr[GRCh37] 14q32.33(105853304_107285437)x4	~6	~5	~5	~5	~6	5~6	5~6	~5	~5	~5	~5	~5
dup(16)(q23.2q24.3)	arr[GRCh37] 16q23.1q24.3(78945304_90155062)x3	50%	+	+	+	+	+	4	+	+	+	+	+
dup(17)(p11.2q25.3)	arr[GRCh37] 17p11.2q25.3(19256657_81041938)x3	+	+	+	+	+	+	+	+	+	+	+	+
amp(18)(p11.32q21.33)	arr[GRCh37] 18p11.32q21.33(136226_61048621)x5-6	5~6	4~6	4~6	4~7	5~6	5~6	5~6	5~6	5~6	5~6	5~6	4~6
+20	arr(20)x3	-	+	-	-	-	-	-	-	-	-	-	-
dup(20)(q11.23q13.33)	arr[GRCh37] 20q11.23q13.33(35758384_62915555)x3	-	-	-	-	-	-	-	-	-	-	-	-
del(22)(q11.22q11.22)x2	arr[GRCh37] 22q11.22(22551349_23268052)x0	+	+	+	+	+	+	+	+	+	+	+	+

Supplementary Table S2. Antibodies used in flow cytometry assays for tumor phenotyping and flow cytometry-based cytotoxicity assays.

**Target**	**Fluoro-chrome**	**Clone**	**Cat nr**	**Company**	**Assay**
LIVE/DEAD Fixable Dead Cell Stain Near-IR	(APC-H7)			Invitrogen	Tumor phenotyping Cytotoxicity
CD45	Krome Orange	J.33	336294	Beckman Coulter	Tumor phenotyping Cytotoxicity
CD20	FITC	L27	345792	BD Biosciences	Tumor phenotyping Cytotoxicity
CD19	PE-CF594	HIB19	562294	BD Horizon™	Tumor phenotyping Cytotoxicity
Kappa	PE		Roy3601	DAKO	Cytotoxicity
Lambda	FITC		CYT-LambF	Emele Bioscience	Cytotoxicity
CD38	FITC	HUMAB003	90002902	BioLegend	Tumor phenotyping
CD10	APC	HI10a	312210	BioLegend	Tumor phenotyping Cytotoxicity
CD38	FITC	Multi-epitope	CYT-38F2	Cytognos	Cytotoxicity
CD138	PE	B-A38	A54190	Beckman Coulter	Cytotoxicity
CD14	BB700	MφP9	566465	BD Biosciences	Cytotoxicity
CD3	BUV395	UCHT1	563546	BD Horizon™	Cytotoxicity
CD56	BUV737	NCAM16.2	564447	BD Horizon™	Cytotoxicity
CD56	PerCP-CY5.5	B159	560842	BD Pharmingen	Cytotoxicity
HLA-DR	BB700	646-6	566480	BD Horizon™	Tumor phenotyping
HVEM	BV711	CW10	743826	BD Optibuild	Tumor phenotyping
Galectin-9	APC	9M1-3	348908	Biolegend	Tumor phenotyping

Supplementary Table S3. Antibodies used in immunoblotting.

Target	Mouse/rabbit	Clone	Catalogus number	Company
c-MYC	Rabbit monoclonal	E5Q6W	18583S	Cell Signaling
BIM	Rabbit monoclonal		2819S	Cell Signaling
BCL-2	Rabbit monoclonal	D5568	4223S	Cell Signaling
PUMA	Rabbit monoclonal		4976S	Cell Signaling
XIAP	Rabbit monoclonal	D278W	14334S	Cell Signaling
Survivin	Mouse monoclonal	6E4	2802S	Cell Signaling
MCL-1	Rabbit monoclonal	D2W9E	94296S	Cell Signaling
B-actin	Mouse monoclonal	8H10D10	3700S	Cell Signaling
Goat anti-rabbit (secondary antibody)	Goat		926-68021	Li-Cor
Goat anti-mouse (secondary antibody)	Goat		926-32210	Li-Cor

Supplementary Figures

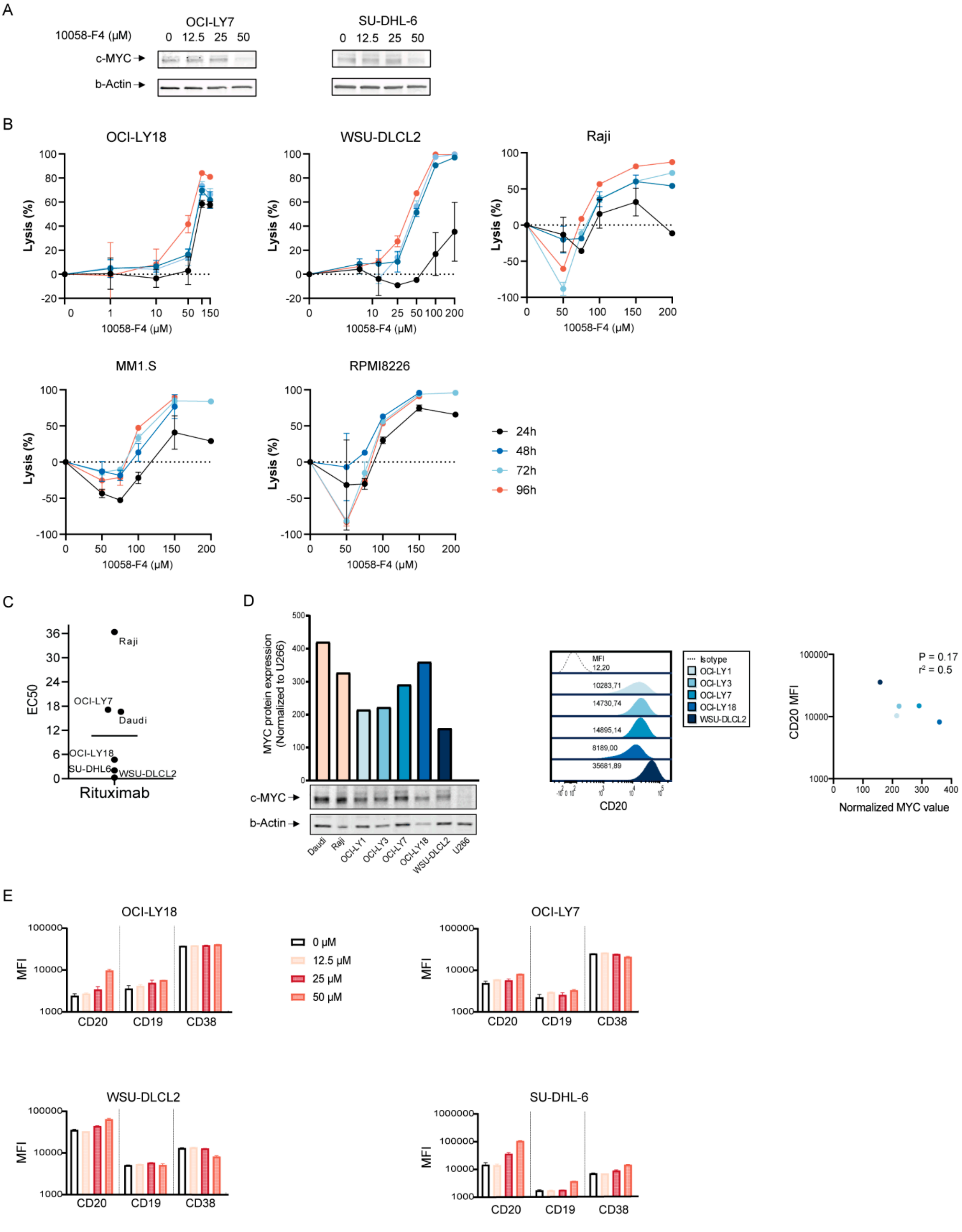

Supplementary Figure S1. Efficacy of MYC inhibitor 10058-F4 and conventional and T-cell-redirecting antibodies in multiple B-cell malignancy cell lines.

(**A**) Additional immunoblot assay of MYC protein and β-actin levels in OCI-LY7 and SU-DHL-6.

(**B**) Cell lines OCI-LY18, WSU-DLCL2, Raji and multiple myeloma (MM) cell lines MM1.S and RPMI8226 were incubated with 0-200 µM 10058-F4 (x-axis) for up to 96 hours (colors). Tumor cell lysis was assessed using a flow cytometry based cytotoxicity assay. Cell viability is calculated based on untreated cells (0 µM).

(**C**) DLBCL and MM cell lines were incubated with rituximab (0-1000 ng/ml, DLBCL) or daratumumab (0-10000 ng/ml, MM) in the presence of PBMCs obtained from healthy donors as effector cells (E:T ratio 40:1) for 16h. Tumor cell lysis was assessed using a bioluminescence based cytotoxicity assay. Cell viability is calculated based on untreated cells. EC50 values (depicted on the y-axis) of monoclonal antibodies rituximab and daratumumab (left) and bispecific T-cell engager blinatumomab (right) as single agents in various cell lines.

(**D**) Left: Immunoblot analysis of MYC protein and β-actin levels at baseline with corresponding bar graph (MYC protein expression normalized to U266 cell line) of Burkitt lymphoma (BL) cell lines Daudi and Raji and diffuse large B-cell lymphoma (DLBCL) cell lines OCI-LY1, OCI-LY3, OCI-LY7, OCI-LY18, WSU-DLCL2. U266 served as a negative control. Right: representative flow-cytometry histogram depicting CD20 expression in BL and DLBCL cell lines. bottom: correlation plot of MYC protein level (Normalized to U266) values (x-axis) versus CD20 expression (as MFI values [y-axis]) in DLBCL cell lines.

(**E**) Bar graphs of CD20, CD19 and CD38 expression in OCI-LY18 (top), WSU-DLCL2 (top), OCI-LY7 (middle) and SU-DHL-6 (bottom) after 24h of treatment with indicated concentrations (0-50 µM) of 10058-F4.

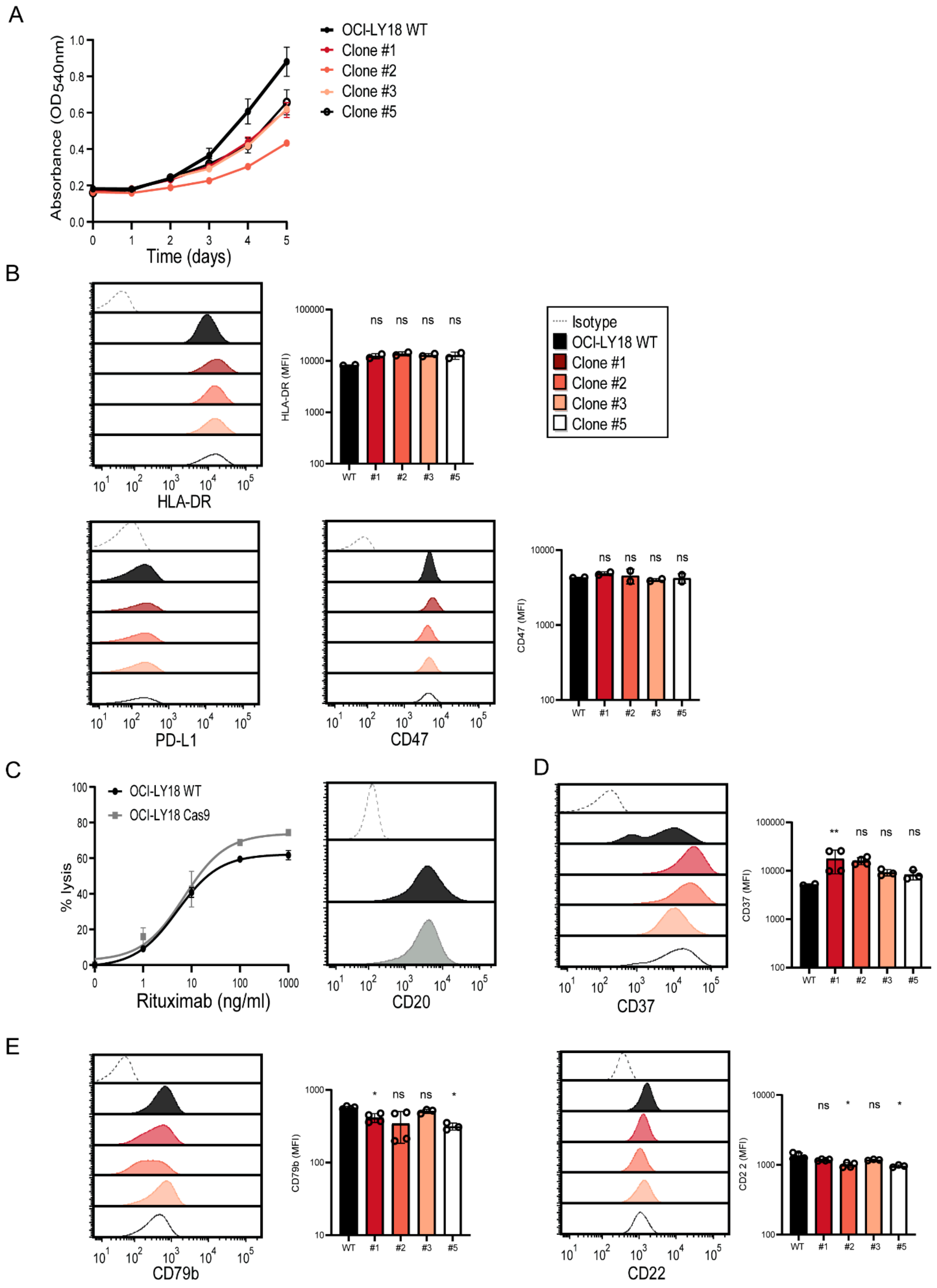

A
Absorbance (OD540nm)
Time (days)
OCI-LY18 WT
Clone #1
Clone #2
Clone #3
Clone #5
B
HLA-DR
HLA-DR (MFI)
ns
Isotype
OCI-LY18 WT
Clone #1
Clone #2
Clone #3
Clone #5
PD-L1
CD47
CD47 (MFI)
WT
#1
#2
#3
#5
C
% lysis
Rituximab (ng/ml)
OCI-LY18 WT
OCI-LY18 Cas9
CD20
D
CD37
CD37 (MFI)
**
E
CD79b
CD79b (MFI)
*
CD22
CD2 2 (MFI)

Supplementary Figure S2. **Characterization of MYC targeted clones.**

(**A**) Proliferation assay of in OCI-LY18 wild type (WT, black) and a selection of 4 representative clones (colored) for 5 days (x-axis) as determined with an MTT-assay. Absorbance values (OD_{540nm}) are depicted on the y-axis. (**B**) Representative flow-cytometry histograms (left) and corresponding mean fluorescent values (MFI) (right) depicting HLA-DR, PD-L1 and CD47 in OCI-LY18 WT (black) and MYC targeted clones (colored). Expression data are representative histograms of 2 technical replicates. (**C**) Cytotoxicity assay of OCI-LY18 WT (black) and OCI-L18 cells lentivirally transduced with Cas9 (OCI-LY18 Cas9, grey) with indicated concentrations of rituximab on the x-axis in the presence of PBMCs obtained from healthy donors as effector cells (E:T ratio 40:1) (left) and representative flow-cytometry histograms depicting CD20 in OCI-LY18 WT (black) and OCI-LY18 Cas9 (grey) (right). Cytotoxicity data are presented as mean ± SEM of at least 3 technical replicates. Cell lysis is calculated within each cell line compared with untreated cells. Expression data are representative histograms of 2 technical replicates. (**D-E**) Representative flow-cytometry histograms (left) and corresponding mean fluorescent values (MFI) (right) depicting of CD37 (D) and CD79b and CD22 (E) in OCI-LY18 WT (black) and MYC targeted clones (colored). * $p < 0.05$; ** $p < 0.01$; ns not significant

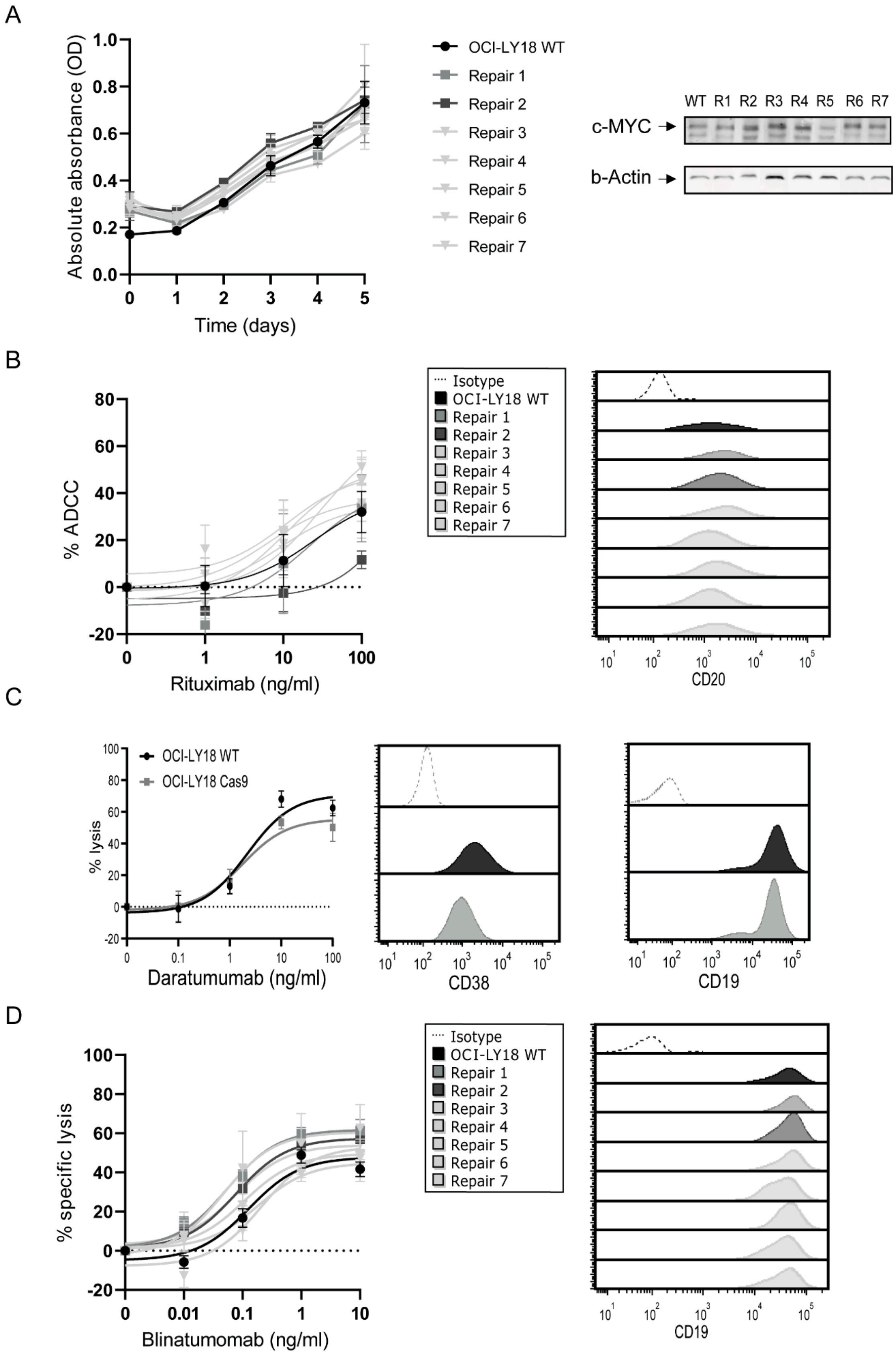
A
Absolute absorbance (OD)
Time (days)
OCI-LY18 WT
Repair 1
Repair 2
Repair 3
Repair 4
Repair 5
Repair 6
Repair 7
WT R1 R2 R3 R4 R5 R6 R7
c-MYC
b-Actin
B
% ADCC
Rituximab (ng/ml)
Isotype
OCI-LY18 WT
Repair 1
Repair 2
Repair 3
Repair 4
Repair 5
Repair 6
Repair 7
CD20
C
% lysis
Daratumumab (ng/ml)
OCI-LY18 WT
OCI-LY18 Cas9
CD38
CD19
D
% specific lysis
Blinatumomab (ng/ml)
Isotype
OCI-LY18 WT
Repair 1
Repair 2
Repair 3
Repair 4
Repair 5
Repair 6
Repair 7
CD19

Supplementary Figure S3. Characterization of repaired MYC subclones.

(**A**) Proliferation assay of in OCI-LY18 wild type (WT, black) and a selection of representative MYC repaired subclones clones (grey) for 5 days (x-axis) as determined with an MTT-assay. Absorbance values (OD_{540nm}) are depicted on the y-axis.

(**B** on the left) OCI-LY18 WT (black) and MYC repaired subclones (grey) were incubated with indicated concentrations of rituximab for 16h for 16h (x-axis) in the presence of PBMCs obtained from healthy donors as effector cells (E:T ratio 40:1). Percentage of tumor cell lysis is depicted on the y-axis. Tumor cell lysis was assessed using a flow cytometry based cytotoxicity assay. Cell lysis is calculated within each cell line compared with untreated cells. Data are presented as mean ± SEM of at least 3 technical replicates. (C on the right) Representative flow-cytometry histogram depicting CD20 expression expression (C) in OCI-LY18 WT (black) and MYC repaired subclones (grey). Expression data are representative histograms of at least 2 technical replicate.

(**C**) Cytotoxicity assay of OCI-LY18 WT (black) and OCI-L18 cells lentivirally transduced with Cas9 (OCI-LY18 Cas9, grey) with indicated concentrations of daratumumab on the x-axis in the presence of PBMCs obtained from healthy donors (E:T ratio 40:1). (left) and representative flow-cytometry histograms depicting CD38 and CD19 in OCI-LY18 WT (black) and OCI-LY18 Cas9 (grey) (right). Details as in (C).

(**D** on the left) OCI-LY18 WT (black) and MYC repaired subclones (grey) were incubated with indicated concentrations of blinatumomab for 24h. (D on the right) Representative flow-cytometry histogram depicting CD19 expression (C) in OCI-LY18 WT (black) and MYC repaired subclones (grey). Details as in B.

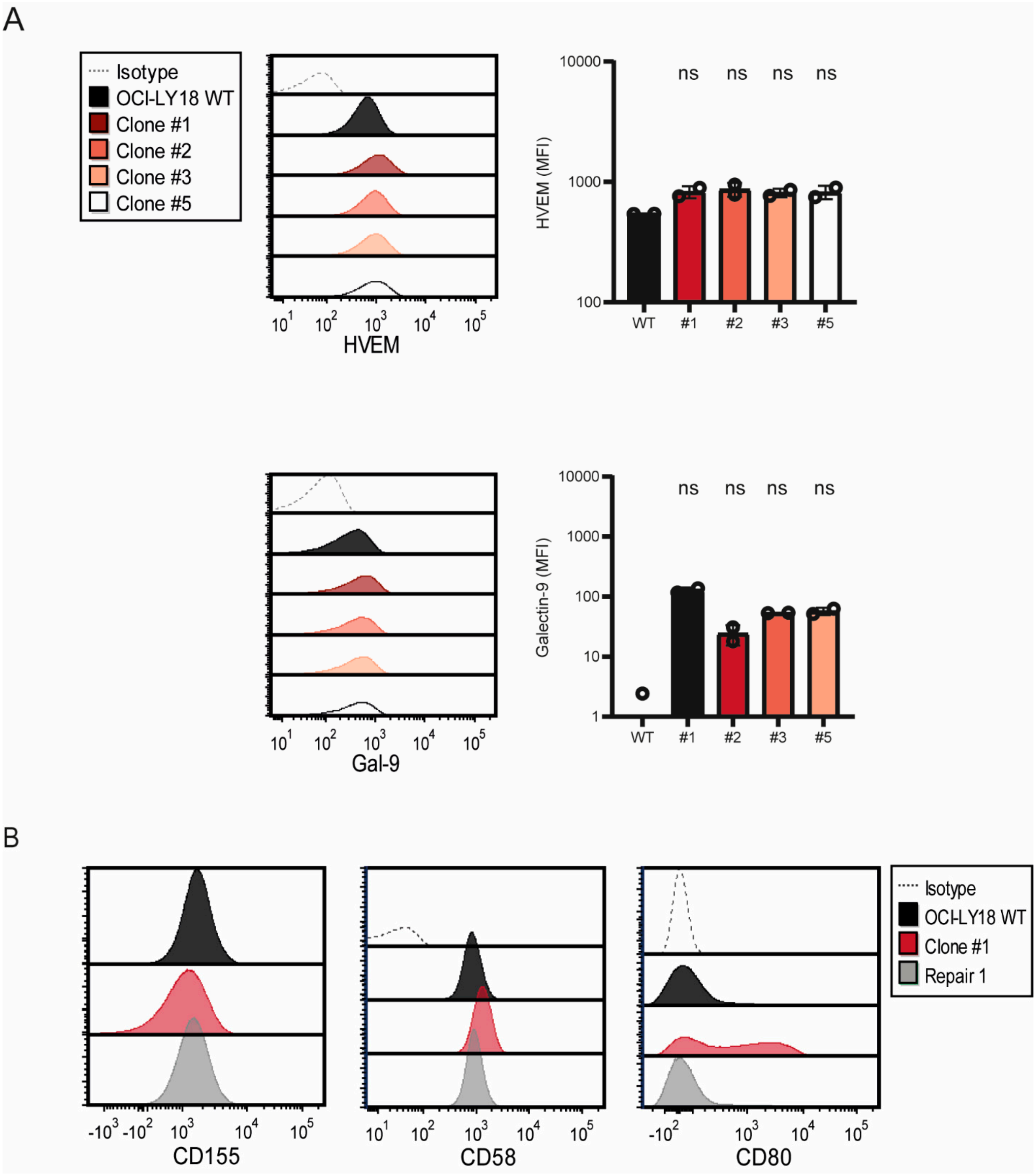

Supplementary Figure S4. Expression of molecules involved in T-cell activation in MYC targeted clones and repaired MYC subclones.

(**A**) Representative flow-cytometry histograms (left) and corresponding mean fluorescent values (MFI) (right) depicting HVEM and Galectin-9 expression in OCI-LY18 wild type (WT, black) and

MYC targeted clones (colored). (**B**) Representative flow-cytometry histograms depicting CD155, CD58, CD80 expression in OCI-LY18 WT (black) and MYC targeted clone #1 (red) and MYC repaired subclones (grey). Expression data are representative histograms of 2 technical replicates.
